# Analysis of risk factors for adjacent superior vertebral pedicle-induced facet joint violation during the minimally invasive surgery transforaminal lumbar interbody fusion: a retrospective study

**DOI:** 10.1186/s40001-015-0174-9

**Published:** 2015-09-24

**Authors:** Zhi-Li Zeng, Long Jia, Wei Xu, Yan Yu, Xiao Hu, Yong-Wei Jia, Jian-Jie Wang, Li-Ming Cheng

**Affiliations:** Department of Spine Surgery, Tongji Hospital, Tongji University School of Medicine, 389 Xincun Road, Shanghai, China

**Keywords:** MIS-TLIF, Facet joint violation, Pedicle screw, Risk factor

## Abstract

**Background:**

The purpose was to explore possible risk factors of facet joint violation induced by adjacent superior vertebral pedicle screw during the minimally invasive surgery transforaminal lumbar interbody fusion (MIS-TLIF).

**Methods:**

A total of 69 patients with lumbar degenerative disease, who underwent MIS-TLIF were retrospectively reviewed. Postoperative computed tomography images were used to assess the facet joint violation. The correlation of facet joint violations with gender, age, body mass index (BMI), the adjacent superior vertebral level, fusion segment numbers, position of screw insertion, straight leg-raising test (SLRT) results, clinical diseases and renal dysfunction were analyzed by Chi-square tests and binary logistic regression analysis.

**Results:**

The incidence of adjacent superior facet joint violations was 25.4 %. Chi-square test showed the patients with age <60 and high BMI (≥30 kg/m^2^) were more prone to have facet joint violations (*P* = 0.007; *P* = 0.006). The single segment fusion presented more facet joint violations than the double segments fusion (*P* = 0.048). The vertebral pedicle screw implant location at L5 showed more facet joint violations compared with that at L3 and L4 (*P* = 0.035). No correlation was found between gender, screw implant position, SLRT results, clinical diseases and renal dysfunction and facet joint violations. Logistic regression analysis revealed that age <60 years (OR: 2.902; 95 % CI 1.227–6.864; *P* = 0.015) and BMI ≥30 kg/m^2^ (OR: 2.825; 95 % CI 1.191–6.700; *P* = 0.018 < 0.05) were significantly associated with facet joint violation.

**Conclusion:**

These results found a high incidence of adjacent superior vertebral facet joint violation in the MIS-TLIF. Age <60 and BMI ≥30 kg/m^2^ might be risk factors of facet joint violation.

Evidence level: Level 4.

## Background

Transforaminal lumbar interbody fusion (TLIF) is prevalent in the management of some spinal disorders that require lumbar fusion [1, 2]. Recently, with development of minimally invasive concept and medical instruments, minimally invasive surgery transforaminal lumbar interbody fusion (MIS-TLIF) has been increasingly accepted due to its advantages in less intraoperative blood loss, weaker intensity of postoperative pain, and shorter hospitalization [3–5].

Recently, increasing concern has been given on the postoperative complications. The most common long-term complication is adjacent segment degeneration. There is evidence that sagittal orientation or tropism at the adjacent segment might be potential risk factors of adjacent segment degeneration [6]. Altered Sagittal balance [7, 8] and deperiostation [9] also contribute to more degeneration of adjacent segment. Biochemical analysis reveals that the lumbar fusion causes increased facet loading that might lead to adjacent segment degeneration [10, 11]. Moreover, it has been established that the adjacent vertebral facet joint violation is a potential risk factor for accelerating the adjacent segment degeneration [12, 13]. Furthermore, one main contributor to the facet joint violations is placement of the pedicle screws [13, 14].

In MIS-TLIF, pedicle screws are inserted percutaneously without direct visualization of the facet joint, and suspected to be responsible for increased facet joint violations and long-term risk of adjacent segment degeneration [15–17]. To date, several studies have compared open and percutaneous pedicle screw placement and explored the risk factors of facet violation. For instance, it has been revealed that minimally invasive pedicle screw placement does not result in increased facet violation compared with open surgery, and higher BMI is a potential contributor to increased facet violation [18]. However, another study argues that percutaneous pedicle screw placement is associated with higher incidences of high-grade facet joint violation relative to open surgery, and several potential risk factors of facet violation are identified, such as age <65, pedicle screw placement at L4 and obesity [19]. Regardless of these controversial viewpoints, exploring the risk factors that contribute to the facet joint violations in MIS-TLIF is very imperative. There are few studies investigating the risk factors of facet joint violations specifically in patients undergoing MIS-TLIF. Therefore, in this study, we retrospectively reviewed the clinical data of 69 patients who underwent MIS-TLIF, and assessed relevant risk factors of facet joint violation caused by adjacent superior vertebral pedicle screw, including gender, age, BMI, the location of adjacent upper vertebral, fusion segment numbers, screw implant location and others.

## Methods

### The patients

**Fig. 1:**
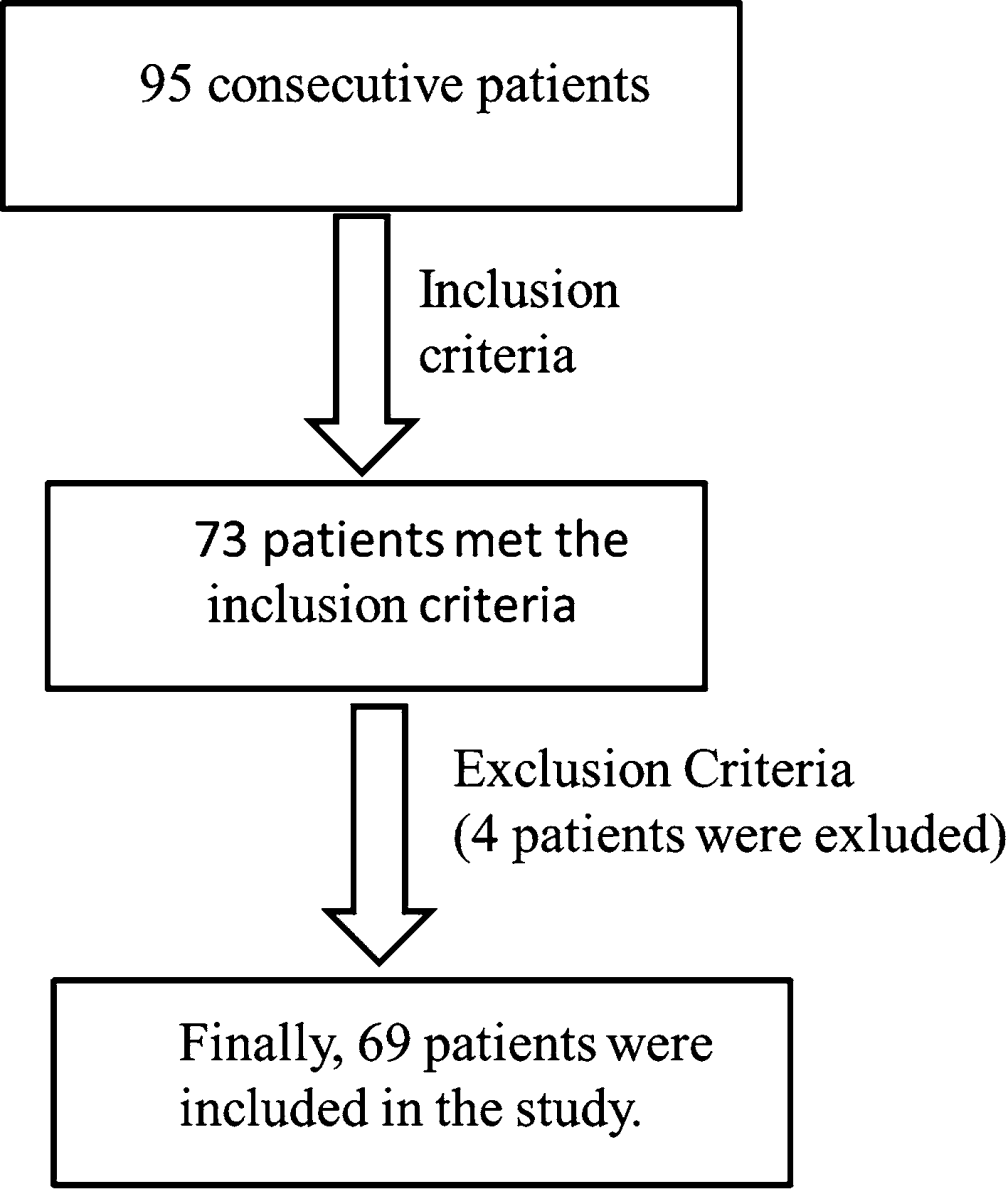
Flow chart of patients’ selection

From December, 2012 to June, 2014, 95 consecutive patients with lumbar degenerative disease (male, 35; female, 34; average age: 54.8 ± 4.8 years) who underwent minimally invasive surgery transforaminal lumbar interbody fusion (MIS-TLIF) were retrospectively reviewed in this study (approved by Tongji Hospital, Tongji University School of Medicine). The surgical indications were patients who had clear lumbocrural pain or fall bilge feeling and invalid improvements after at least 3 months of conservative treatment. Of the 95 patients, 73 patients met the inclusion criteria: patients suffered from lumbar degenerative disease with lumbar disc herniation or lumbar spinal stenosis or lumbar spondylolisthesis or endplate Modic changes [20]. These diseases were confirmed by preoperative computed tomography (CT) and magnetic resonance imaging (MRI). The exclusion criteria were as follows: presence of obvious pedicle screw position deviation or even needing a second revision surgery because of non-standard surgical manipulation; degenerative lumbar scoliosis; spinal tumor. Then, 4 patients were excluded. Finally, 69 patients were included in the study with complete medical records and follow-up data (Fig. 1). Among the patients, 45 patients were with hip or unilateral lower limb radiation pain, 20 patients were with bilateral lower limb radiation pain, 25 patients were with positive results of straight leg-raising test (SLRT) and strengthen test, and 3 patients were with urine dysfunction.

Preoperative CT and MRI examination showed obvious lumbar disc herniation and (or) lumbar spinal stenosis in 48 cases, lumbar spondylolisthesis in 9 cases and endplate Modic changes in 12 cases. The study protocol was approved by local ethics committee. Informed consent was obtained from each patient prior to the study. The operation was performed by two deputy or chief surgeons who had more than 15-year experience in orthopedic surgery. There were 62 cases with single segment fusion and 7 cases with double segments fusion. The position of adjacent superior vertebral pedicle screw insertion in these cases was detailed as follows: L3, 4 cases; L4, 45 cases; L5, 20 cases. In addition, 69 pedicle screws were placed on the left and the right side of the spine of the patients, respectively.

### Surgical technique

**Fig. 2:**
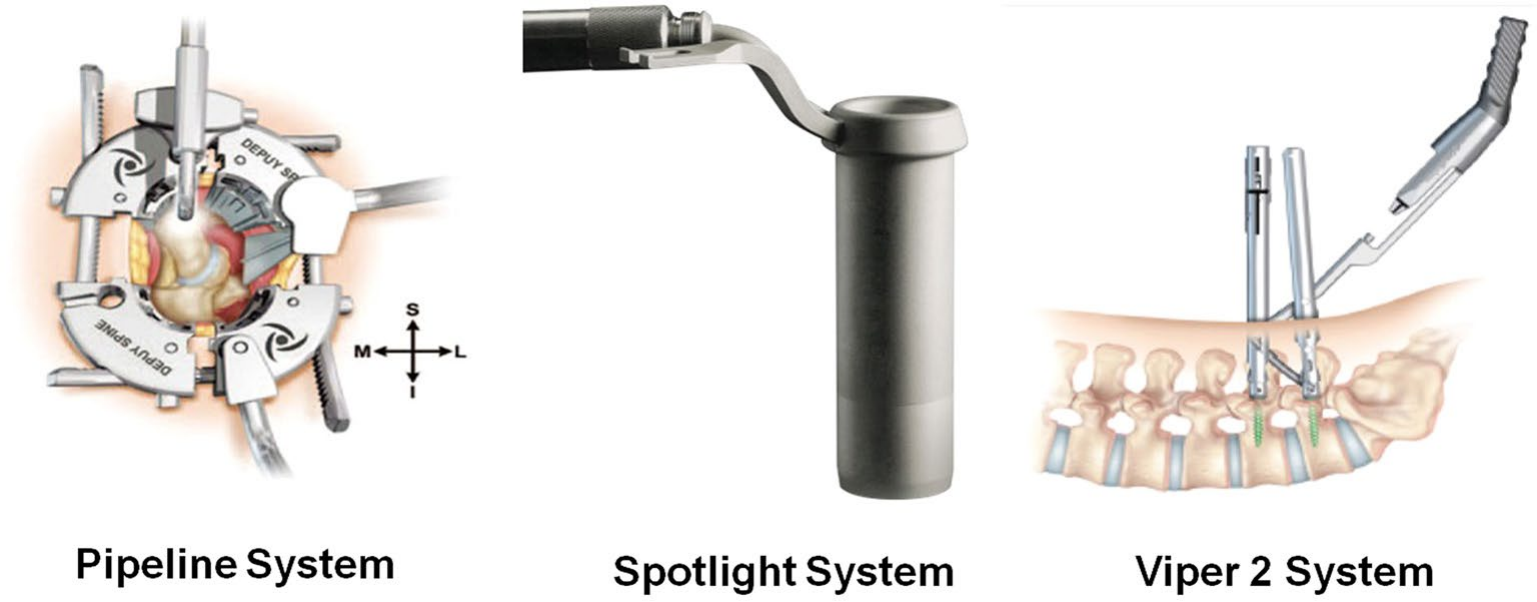
The instrument used in the procedure of MIS-TLIF

Patients were kept in prone position following general anesthesia and their abdomen was suspended and pressure parts were with pad. C arm fluoroscopy was used to determine available surgical space. A 2- to 3-cm incision was made approximately 2.5 cm lateral to the midline to cut skin and muscular fasciae. After inserting the dilators step by step, Pipeline working channel (Johnson & Johnson Company, NY, USA, Fig. 1) was placed into and fixed by dilators, or directly using Spotlight working channel (Johnson & Johnson Company, NY, USA, Fig. 2). Then, the local soft tissue was removed to expose vertebral plate edges and facet joint. The decompression was performed to expose dural sac, the central canal, lateral crypt and nerve root canal after removing part of vertebral plate, ligamentum flavum and facet joints. After thoroughly removing intervertebral disc and cartilage endplate, local autologous bone was implanted into intervertebral space, and then single suitable height of intervertebral fusion was placed. For bilateral decompression or more, the same method was performed to deal with the contralateral and other spaces. Under the guidance of C arm fluoroscopy, placement of percutaneous pedicle screws was performed using Viper2 system (Johnson & Johnson Company, NY, USA, Fig. 1) and percutaneous rod was also placed using the instruments and pre-locked. Drainage tube was removed 24–36 h postoperatively. At 3 days postoperatively, the patients were examined with lumbar X-ray and CT to confirm the position of lumbar fusion instruments and internal fixation, and to evaluate the facet joint violation. Moreover, the patients were encouraged to have activities out of bed under waist protection. Waist torsion and bending activities were prohibited within 3 months under waist protection.

### Radiographic evaluation and observation index

**Fig. 3:**
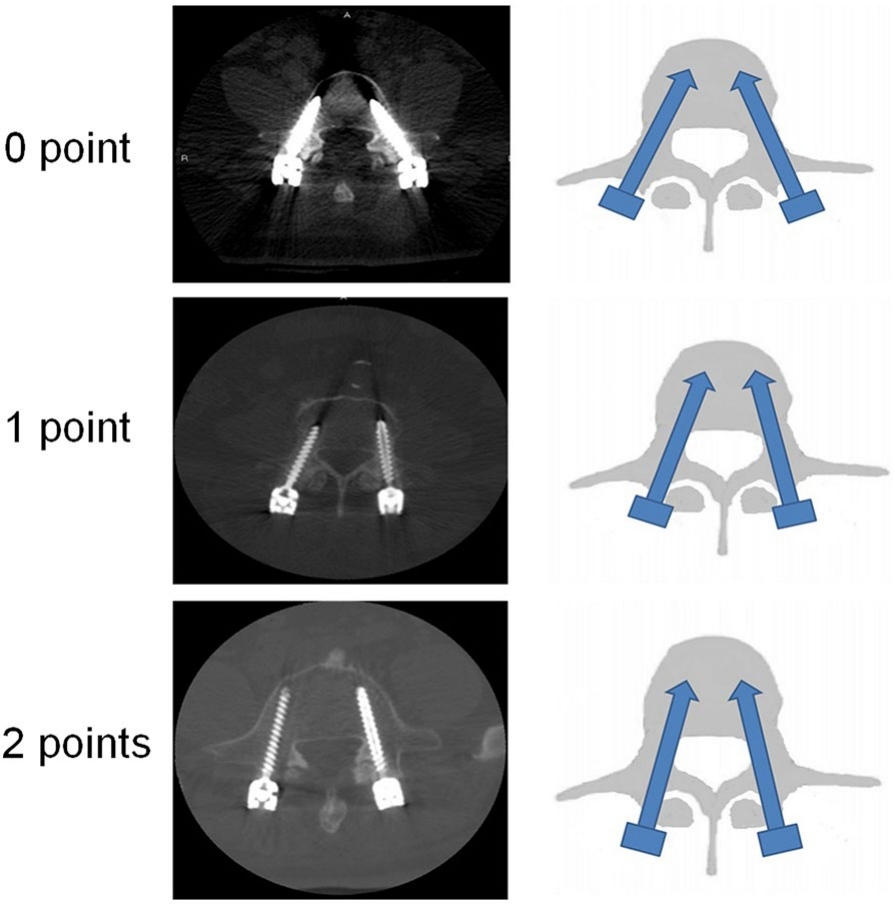
The CT images and corresponding mode pattern of location relationship between vertebral pedicle screw (total number: 138) and facet joints. No point indicated the vertebral pedicle screw clearly avoids the facet joint, one point indicated the vertebral pedicle screw head is either in contact with or suspected to has invaded the facet joint, and two points indicate the screw has clearly invaded the facet joint

All the patients were examined postoperatively by lumbar spinal 64-row CT thin layer scanning with 0.5 mm slices. The CT images were assessed by Picture Archiving and Communication Systems. The evaluation standard was according to Seo taxonomy which developed a point system to evaluate the facet joint violations on patients’ CT scan. Specifically, no points were given when the vertebral pedicle screw clearly avoided the facet joint; one point was given when the vertebral pedicle screw head was either in contact with or suspected to have invaded the facet joint; two points were given when the screw had clearly invaded the facet joint (Fig. 3) [21]. The facet violation grade was assessed independently by two surgeons who were blinded to the clinical diseases of patients. If their results are different, the senior author determined the facet violation grade finally. The inter-observer reliability was calculated according to the kappa statistics (kappa coefficient = 0.65). In addition, X-ray examination was also performed to study the facet violation of the patients operatively.

### Statistics analysis

All analyses were conducted using SPSS software, version 17.0. Gender, age, BMI, adjacent superior vertebral level, fusion segment numbers, screw implant location, SLRT results, clinical diseases and renal dysfunction were compared between patient with or without facet joint violations using Chi-square tests. Binary logistic regression analysis was performed to identify significant risk factors of facet joint violation. *P* < 0.05 was considered to be significant.

## Results

**Fig. 4:**
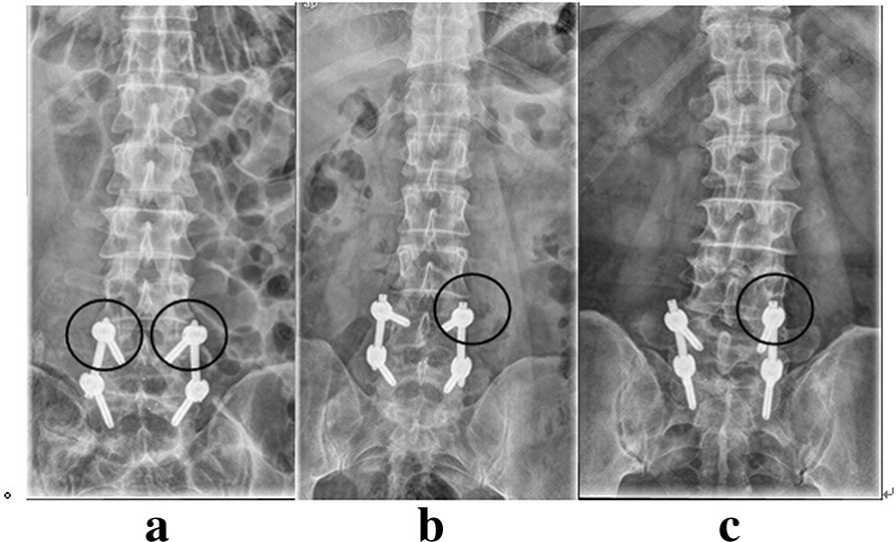
Postoperative X-ray images of patients. **a** X-ray image of 0 point facet joint violation; **b** X-ray image of one point facet joint violation; **c** X-ray image of two point facet joint violation

The 69 patients successfully underwent the MIS-TLIF. The postoperative CT scans showed the location relationship between vertebral pedicle screw (total number: 138) and facet joint: no point, 74.6 % (*n* = 103); one point, 16.0 % (*n* = 22); two points (*n* = 13), 9.4 %. It showed that the incidence of facet joint violations (≥one point) was 25.4 % in these patients. The exemplary X-ray images for no point, one point and two points were exhibited in Fig. 4.

Chi-square test revealed that (Table 1) the patients who were <60 years old, with high BMI (≥30 kg/m^2^) were more prone to have facet joint violations (*P* = 0.007; *P* = 0.006). The single segment fusion presented more facet joint violations than the double segments fusion (*P* = 0.048). The pedicle screw implant at L5 showed more facet joint violations compared with those at L3 and L4 (*P* = 0.035). However, the difference of gender, screw implant location, results of SLRT, clinical diseases and renal dysfunction was not significant between the patients with facet joint violations (1 and 2 points) and the patients without facet joint violation (0 point) (*P* = 0.493; *P* = 0.328; *P* = 0.177; *P* = 0.942; *P* = 0.983).

**Table 1:** Comparison of gender, age, body mass index (BMI), vertebral level, fusion segment numbers, screw implant location, result of straight leg-raising test, clinical diseases and renal dysfunction between patient with or without facet joint violations

Factors	Screw (*n*)	Location relationship between vertebral pedicle screw and facet joint (*n*)		*P* value
		0 Point	1 + 2 Point	
Gender				0.493
Male	70	54	16 (11 + 5)	
Female	68	49	1 (11 + 8)	
Age				0.007*
<60	82	68	14 (10 + 4)	
≥60	56	35	21 (12 + 9)	
BMI				0.006*
<30 kg/m^2^	86	71	15 (10 + 5)	
≥30 kg/m^2^	52	32	20 (12 + 8)	
Adjacent superior vertebral level				0.035*
L3	8	8	0	
L4	90	69	21 (13 + 8)	
L5	40	26	14 (9 + 5)	
Fusion segment				0.048*
Single	124	89	35 (22 + 13)	
Double	14	14	0	
Implant location				0.328
Left	69	49	20 (13 + 7)	
Right	69	54	15 (9 + 6)	
Straight leg-raising test				0.177
Negative result	88	69	19 (12 + 6)	
Positive result	50	33	17 (10 + 7)	
Clinical diseases				0.942
Lumbar disc herniation and spinal stenosis	96	71	25 (14 + 11)	
Lumbar spondylolisthesis	24	18	6 (4 + 2)	
Endplate Modic changes	18	14	4 (3 + 0)	
Renal dysfunction				0.983
No	132	98	34 (22 + 12)	
Yes	6	5	1 (0 + 1)	

Logistic regression analysis was further performed to determine whether age <60 years old, BMI ≥30 kg/m^2^, single segment fusion and pedicle screw implant at L5 were risk factors of facet joint violation. Table 2 shows that relative to patients ≥60 years old, patients younger than 60 years old have 2.902 increased odds of experiencing facet joint violation (95 % CI 1.227–6.864; *P* = 0.015 < 0.05). Besides, patients with BMI ≥30 kg/m^2^ has 2.825 increased odds of experiencing facet joint violation (95 % CI 1.191–6.700; *P* = 0.018 < 0.05) compared to patients with BMI <30 kg/m^2^. Single segment fusion and pedicle screw implant at L5 were not significantly associated with facet joint violation (*P* = 0.998; *P* = 0.071).

**Table 2:** Result of logistic regression analysis

	OR	95 % CI	*P* value
Age >60 years old	2.902	1.227–6.864	0.015
BMI >30 kg/m^2^	2.825	1.191–6.700	0.018
Single segment fusion	0.000	0.000	0.998
Pedicle screw implant at L5	2.177	0.935–5.071	0.071

*OR* odd ratio, *CI* confidence interval

## Discussion

The current study investigated the risk factors of facet joint violations in patients undergoing MIS-TLIF. The results indicated that the incidence of facet joint violations was 25.4 %. Moreover, the study found that age <60 years and high BMI (≥30 kg/m^2^) were suggested to be independent risk factors for facet joint violations. Gender, vertebral level, fusion segment numbers, screw implant location, result of SLRT, clinical diseases and renal dysfunction were not significantly associated with facet joint violations.

Facet joint is an important structure to maintain the stability of lumbar spine motion segments. The paired facet joint with lumbar intervertebral disc constitute the lumbar complex which is responsible for the spine movement, stability, torsion and load-bearing ability [22]. Facet joint violations can destruct the spine stability and further accelerate the development of adjacent segment degeneration [23]. Park et al. [14] have found that compared with other forms of instrumentation or with no instrumentation, transpedicular instrumentation is prone to cause symptomatic adjacent segment disease, and that the possible risk is the adjacent facet joint violations during the pedicle screw placement. Moreover, a cadaveric study also indicates that the complications are more serious when the superior facet joints were violated by the placement of pedicle screw [24]. Therefore, in this study, the results showed that the rate of facet joint violations with percutaneous pedicle screw insertion was 25.4 %, which was not consistent with the previous studies in which the incidence of facet joint violation displays wide variation and ranges from 3.2 to 50 % [19, 25, 26]. One possible contributor to the inconsistent results is the varied definition and assessment methods of facet joint violations in different studies. Moreover, the sample was relatively small in this study which might be another reason responsible for the inconsistent results.

Previously, several studies have investigated the potential risk factors for the joint violations. A retrospective study performed by Ranjith Babu et al. has demonstrated that patients’ age <65 and obesity contribute to the increased difficulty in avoiding the facet joint violations [19]. BMI >29.9 is a potential contributor to increased facet violation [18]. Similarly, in the present study, the logistic regression analysis found that patients younger than 60 years old, with BMI ≥30 kg/m^2^ were independent risk factors of facet joint violations in the MIS-TLIF. The greater skin elasticity and stronger muscle of younger patients may make it more difficult to get the tactile sensation of the facet and to achieve an appropriate entry site. Moreover, it has been reported that clear radiological intraoperative images are not easy to be observed from the obese patients [16, 27], and that the hypertrophic tissue in obese patients increases the distance from the skin to the spine. The above factors make it more difficult to achieve an ideal entry site with appropriate angulation and further decrease the accuracy of percutaneous pedicle screws placement. However, Park et al. [15] have found no significant relationships between patients’ age, BMI and the incidence of facet joint violations. These controversies are needed to be further investigated.

In addition, facet joint violations caused by the placement of pedicle screw are more frequent at the L4, L5 pedicel level than at the L3. The results from Park et al.’s research indicate that violations occur more frequently at the cranial pedicle screws of L5 pedicle than at other pedicels [15]. In the current study, the Chi-square test results also indicated that the incidence of facet joint violations at L5 pedicle was significantly higher than that at the L3 and L4 pedicel levels. The possible explanation is that the facet joint and the caudal portions of the laminae are more toward the frontal plane at L5-S1 level than other levels, which may increase the difficulty of percutaneous screws placement [28]. Moreover, the increased lordosis and paravertebral muscles at the L5-S1 level may also contribute to the increased facet joint violation [29]. Besides, a retrospective study of Moshirfa et al. indicates that a higher incidence of superior-level facet joint violation is observed in single-level fusion compared with that in multiple-level fusion [30]. However, Park et al. [15] have found no correlation between the number of fused segment and facet joint violation. In this study, the Chi-square test also found the single segment fusion was more prone to experience facet joint violation than the double segments fusion. The pedicle screws insertion with single segment fusion in this study was mostly at L5 level where the facet joint violation was more difficult to avoid. It seemed to provide a rational explanation for the results of this study. However, the logistic regression analysis revealed that single segment fusion and pedicle screws insertion at L5 were not significantly associated with the facet joint violation. Further studies of large sample size were needed to validate the results. Meanwhile, Moshirfa et al. [30] found that the screw implant at the left side was prone to cause facet joint violation. Conversely, the present results indicated no significant difference of facet joint violation between the screw placement at the left side and at the right side (left vs. right), which might be attributed to the assistance of C arm fluoroscopy. These controversies and assumption are worthy to be further investigated.

The study is presented with several limitations. First, the sample size of the study was small. Therefore, larger and multiple-center studies are needed in future study to confirm the results. Second, due to the limited duration of follow-up, the correlation between adjacent superior pedicle facet joint violation and late clinical outcome was incapable to be investigated, which will be a focus in the future study. In addition, the association between preoperative lumbar degeneration and facet joint violation was also an intriguing research direction.

## Conclusion

The results from this retrospective study found a high incidence of adjacent superior vertebral facet joint violation in the MIS-TLIF. Age <60 years old and BMI ≥30 kg/m^2^ were independent risk factors of facet joint violation.
